# Improved Survival and Hematopoietic Differentiation of Murine Embryonic Stem Cells on Electrospun Polycaprolactone Nanofiber 

**DOI:** 10.22074/cellj.2016.3835

**Published:** 2016-01-17

**Authors:** Nima Dehdilani, Karim Shamsasenjan, Aliakbar Movassaghpour, Parvin Akbarzadehlaleh, Bahram Amoughli Tabrizi, Hamed Parsa, Fatemeh Sabagi

**Affiliations:** 1 Hematology and Oncology Research Center, Tabriz University of Medical Sciences, Tabriz, Iran; 2 Iran Blood Transfusion Research Center, High Institute for Research and Education in Transfusion Medicine, Tehran, Iran; 3 Department of Pharmaceutical Biotechnology, Faculty of Pharmacy, Tabriz University of Medical Sciences, Tabriz, Iran; 4 Department of Clinical Sciences, Faculty of Veterinary Medicine, Tabriz Branch, Islamic Azad University, Tabriz, Iran

**Keywords:** Mouse Embryonic Stem Cells, Hematopoietic Stem Cells, Nanofiber

## Abstract

**Objective:**

Three-dimensional (3D) biomimetic nanofiber scaffolds have widespread ap- plications in biomedical tissue engineering. They provide a suitable environment for cel- lular adhesion, survival, proliferation and differentiation, guide new tissue formation and development, and are one of the outstanding goals of tissue engineering. Electrospinning has recently emerged as a leading technique for producing biomimetic scaffolds with mi- cro to nanoscale topography and a high porosity similar to the natural extracellular matrix (ECM). These scaffolds are comprised of synthetic and natural polymers for tissue engi- neering applications. Several kinds of cells such as human embryonic stem cells (hESCs) and mouse ESCs (mESCs) have been cultured and differentiated on nanofiber scaffolds. mESCs can be induced to differentiate into a particular cell lineage when cultured as em- bryoid bodies (EBs) on nano-sized scaffolds.

**Materials and Methods:**

We cultured mESCs (2500 cells/100 µl) in 96-well plates with knockout Dulbecco’s modified eagle medium (DMEM-KO) and Roswell Park Memorial Institute-1640 (RPMI-1640), both supplemented with 20% ESC grade fetal bovine serum (FBS) and essential factors in the presence of leukemia inhibitory factor (LIF). mESCs were seeded at a density of 2500 cells/100 µl onto electrospun polycaprolactone (PCL) nanofibers in 96-well plates. The control group comprised mESCs grown on tissue cul- ture plates (TCP) at a density of 2500 cells/100 µl. Differentiation of mESCs into mouse hematopoietic stem cells (mHSCs) was performed by stem cell factor (SCF), interleukin-3 (IL-3), IL-6 and Fms-related tyrosine kinase ligand (Flt3-L) cytokines for both the PCL and TCP groups. We performed an experimental study of mESCs differentiation.

**Results:**

PCL was compared to conventional TCP for survival and differentiation of mESCs to mHSCs. There were significantly more mESCs in the PCL group. Flowcyto- metric analysis revealed differences in hematopoietic differentiation between the PCL and TCP culture systems. There were more CD34+(Sca1+) and CD133+cells subpopulations in the PCL group compared to the conventional TCP culture system.

**Conclusion:**

The nanofiber scaffold, as an effective surface, improves survival and differentiation of mESCs into mHSCs compared to gelatin coated TCP. More studies are necessary to understand how the topographical features of electrospun fibers af- fect cell growth and behavior. This can be achieved by designing biomimetic scaffolds for tissue engineering.

## Introduction

Tissue engineering provides a foundation for devising *in vitro* physiological models to studydisease pathogenesis and develop molecular therapeutics (1). Recently, reports demonstrate that both morphological and biological functions can be profoundly governed by three-dimensional (3D) geometry (2,7). Engineering a 3D cellular microenvironment to capture complex 3D tissue physiology *in vitro* (8,9) can aid in mechanistic studies (10) or drug development (11,12). 

The interaction of cells with the extracellular matrix (ECM) plays an important role in governing cell differentiation. For example, the developmental fate of embryonic stem cells (ESCs) is determined not only by soluble factors but also by physical interactions with the surrounding ECM and/or molecules embedded within this ECM (13). Polymeric scaffolds, used as an analogue to the ECM in tissue engineering, have been shown to influence ESCs differentiation and organization (4). Therefore, the design of scaffolds which most closely resembles the native ECM is expected to play a critical role in developing 3D models for hematopoiesis. 

ESCs, derived from the inner cell mass of the pre-implantation blastocyst, are pluripotent and have the potential for unlimited expansion and targeted differentiation (14,15). Maintenance of pluripotency in mESCs depends on the leukemia inhibitory factor (LIF) cytokine’s activation of a heterodimeric complex composed of gp130 and the low-affinity LIF receptor (16). 

A number of authors have reported the ability of ESCs to differentiate into cardiomyocytes (17), hematopoietic cells (18), endothelial cells (19,20), neurons (21,22), chondrocytes (23,24), adipocytes (25,26), hepatocytes (27,28) and pancreatic islets (29). 

Hematopoietic differentiation of ESCs can be performed with different techniques that include the use of feeder layers, embryoid body (EB) formation, cytokine cocktails, and/or a combination of these techniques (30) as well as siRNAs and ectopic gene technology (31,32). Differentiation of ESCs depends on the synergetic effect of proper molecular stimuli and the specific physical structure of the ESC culture condition. Development of a hematopoietic lineage in mouse EBs (mEBs) has been stimulated by interleukin-6 (IL-6) alone (33) or in combination with IL-3 and stem cell factor (SCF) (34). Hematopoietic differentiation of EBs can be achieved by other ap proaches that use different biomaterial structures such as highly porous, tantalum-based scaffolds. These scaffolds have been shown to improve hematopoietic differentiation compared to tissue culture plates (TCP) (35). In addition, several reports have described culturing of ESCs on 3D scaffolds that led to ESC differentiation based on the composition of the scaffold (4,35,36). In this study, we combined ESC biology and biomaterials technology in order to develop an *in vitro* early hematopoietic differentiation model using mESCs seeded into polycaprolactone (PCL). 

## Materials and Methods

In this experimental study, mouse ESCs (mESCs, C571) and PCL were gifted from the Stem Cell Technology Research Center (Iran) based on the Ethical Committee approval of the Faculty of Veterinary Medicine, Tabriz Branch, Islamic Azad University, Tabriz, Iran. Knockout Dulbecco’s modified eagle medium (DMEM-KO), Roswell Park Memorial Institute-1640 (RPMI-1640), Iscove’s modified Dulbecco’s media (IMDM) and ESC grade fetal bovine serum (FBS) were obtained from Sigma. Sterile gelatin (0.1%), LIF, L-glutamine (L-Glu), 2-mercaptoethanol (2-ME), nonessential amino acids (NEAA), penicillin and streptomycin (Pen/Strep) were purchased from Gibco (USA). SCF, IL-3, IL-6 and Fms-related tyrosine Kinase ligand (Flt3) ligand (FL) were obtained from Genescript (USA). 

### Characteristics of polycaprolactone

The molecular weight of PCL was 80 KDa. The average thickness of the PCL scaffolds was 100 µm with a porosity of ~88%, an average pore size of 30 µm and average fiber diameter [based on>100 scanning electron microscope (SEM) measurements] of 690 nm. 

### Preparation of nanofiber scaffolds for cell culture

The nanofiber scaffolds were sterilized by immersion in a 70% ethanol solution for a period of 60 minutes followed by exposure to ultra violate (UV) radiation for 60 minutes. Thereafter, the scaffolds were soaked overnight in culture medium prior to cell seeding in order to facilitate protein adsorption and cell attachment on the nanofiber surface. 

### Mouse embryonic stem cells culture

mESCs were cultured according to a modified protocol based on previously reported methods from Shen and Qu (37). Briefly, gelatin (0.1%) in phosphate-buffered saline (PBS) was poured into 96-well culture plates. The plates were incubated for 30 minutes at room temperature. The excess gelatin solution was removed by aspiration and the plates were allowed to air dry for 20 minutes at room temperature.

mESCs were suspended in 25 cm^2^ gelatin-coated TCP at a density of 1-3×10^5^ cells/10 ml in DMEMKO with 20% (v/v) heat-inactivated FBS and essential factors [100 U/ml penicillin, 100 mg/ml streptomycin, 2-ME (2 mM), NEAA (0.1 mM), L-Glu (2 mM) and 10 ng/ml LIF]. Cells were incubated under conditions described above with a change of media every 24 hours until the mESCs were approximately 70% confluent. Then, mESCs were trypsinized (0.25% trypsin) and suspended at a density of 2500 cells/100 μl (in 96-well plates) in DMEM-KO and RPMI-1640 (both supplemented with 20% FBS) with essential factors. mESCs cultured on PCL were seeded onto PCL that had been secured into 96-well plates (Corning Inc., USA) at a density of 2500 cells/100 μl. mESCs grown on TCP at the same density were used as control cells. Cells cultured on PCL were harvested by an enzyme-free cell dissociation solution (Gibco, USA). After 48 and 96 hours, we performed trypan blue cell staining and counted the viable cells.

### Formation of embryoid bodies in vitro

The ESCs were grown on 96-well plates and maintained in an undifferentiated state using DMEM-KO (20% FBS) with 10 ng/ml LIF. At day 7, the wells were examined under an inverted microscope and healthy, round-shaped EBs were counted. 

### Differentiation stage

To initiate mESCs differentiation into mouse hematopoietic stem cells (mHSCs), we modified a protocol based on previously reported methods from shen and Qu (37). Prior to the differentiation stage, we performed a two day pre-differentiation stage using IMDM (supplemented with 30% FBS) with 10 ng/ml LIF. The medium was changed daily. Differentiation was performed for 7 days. Briefly, mEBs were counted and reseeded in 12-well plates under PCL and TCP conditions in IMDM with a hematopoietic lineage cytokine cocktail that included SCF (20 ng/ml), IL-3 (20 ng/ml), IL-6 (2 ng/ml) and FL (20 ng/ml). The media was changed every 2 days. After 7 days, differentiated mESCs were evaluated by flowcytometry to detect CD34 and CD133 cell surface marker levels. 

### Flowcytometry

On day 0 of the EBs culture, the medium was aspirated followed by washing with 1 mL of PBS. Then, cells were disassociated by trypsin treatment, and re-suspended in PBS. Surface marker labeling was accomplished using SSEA1-PE, CD117-FITC, CD34-PE and CD133-FITC specific antibodies. After 7 days from the onset of the differentiation stage, the medium was aspirated from each well followed by washing with 1 mL of PBS. The differentiated mESCs were removed from each well, centrifuged, and resuspended in PBS. Extracellular antigen labeling was accomplished using CD34 and CD133 for cells and analyzed by flowcytometry. Cellular fluorescence was detected using a FACSCalibur flowcytometer (Becton and Dickinson, USA). As a control, cells stained with isotype monoclonal antibody were used to check for nonspecific background staining. 

### Statistical analysis

For optimization of maintenance medium, we performed one-way ANOVA analysis. The t test was used to compare PCL and TCP data. The data were presented as mean ± SE. P<0.01 was considered significant. Each experiment was replicated at least three times. 

## Results

### Comparison of polycaprolactone and tissue culture plate conditions for mouse embryonic stem cells grown in knock-out DMEM and Roswell Park Memorial Institute-1640 with 20% fetal bovine serum

**Fig.1:**
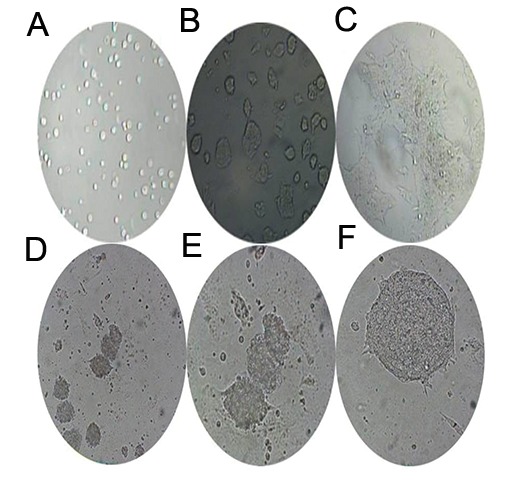
Microscopic images of mouse embryonic stem cells (mESCs) grown in knockout Dulbecco’s modified eagle medium (DMEM-KO) with 20% fetal bovine serum (FBS). A. mESCs after immediate culture (×4), B. mESCs after 48 hours (×10), C. mESCs after 96 hours (×10), D. 6-day old mouse embyroid bodies (mEBs, ×10), E. 6-day old mEBs (×20) and F. 8-day old mEBs (×20).

**Fig.2:**
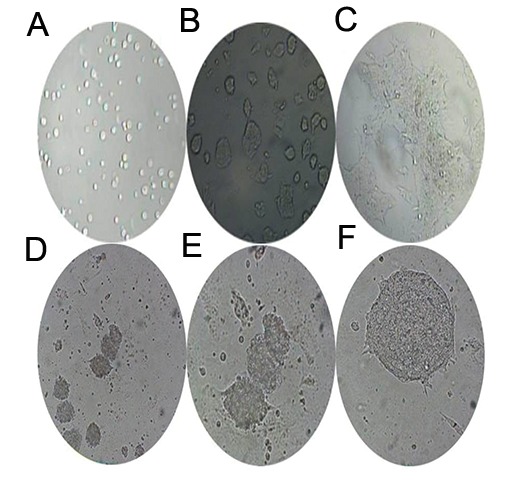
Comparison of polycaprolactone (PCL) and tissue culture plate (TCP) conditions for mouse embryonic stem cells (mESCs) grown in knockout Dulbecco’s modified eagle medium (DMEM-KO) and Roswell Park Memorial Institute-1640 (RPMI-1640) with 20% fetal bovine serum (FBS). mESCs were cultured on PCL along with DMEM-KO or RPMI-1640 and compared to TCP with the same conditions for 48 and 96 hours. *; P<0.01 is significant.

cells grown in knock-out DMEM and Roswell Park Memorial Institute-1640 with 20% fetal bovine serum mESCs were seeded onto PCL that had been secured into 96-well plates and cultured in DMEMKO or RPMI-1640 supplemented with LIF and 20% FBS for 48 and 96 hours. mESCs grown in the TCP were used as the control (Fig .1). mESCs were collected after 48 and 96 hours and stained with trypan blue. Viable mESCs were counted under an inverted microscope. Our results showed that significantly more mESCs grown PCL in both DMEM-KO and RPMI-1640 compared to TCP conditions (P<0.05). There were significantly more mESCs cultured on PCL in DMEM-KO compared to the other conditions (Fig .2,P<0.01). 

### Comparison of the mouse embryonic stem cell population expressing surface markers under polycaprolactone and tissue culture plate conditions

**Fig.3:**
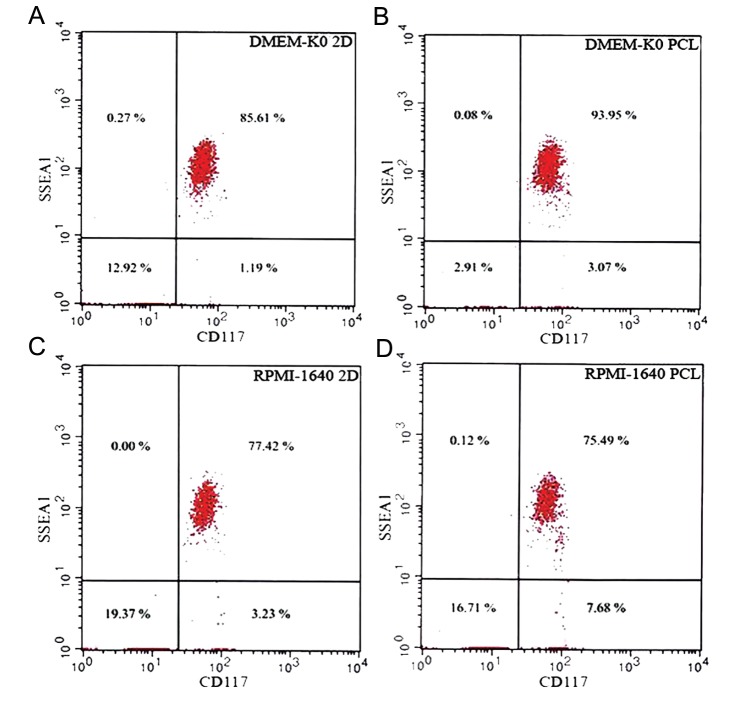
Flowcytometric analysis of the mouse embryonic stem cell (mESC) surface markers under different culture conditions. Flowcytometric analysis was performed for the mESC markers using SSEA1-PE and CD117-FITC antibodies. A. Cell population that expressed SSEA-1 and CD117 in knockout Dulbecco’s modified eagle medium (DMEM-KO) grown in tissue culture plates (TCPs), B. Cell population that expressed SSEA-1 and CD117 in DMEM-KO polycaprolactone (PCL), C. Cell population that expressed SSEA-1 and CD117 in Roswell Park Memorial Institute-1640 (RPMI-1640) TCP and D. Cell population that expressed SSEA-1 and CD117 in RPMI-1640 PCL.

We cultured mESCs in PCL and TCP conditions for 7 days in DMEM-KO or RPMI-1640 supplemented with 20% FBS in order to develop mEBs. Next, we assessed for cells that expressed SSEA1, CD117, CD34 and CD133. Our results showed that compared to the TCP conditions, cells maintained on PCL had significantly higher cell populations that expressed SSEA1 and CD117 cell surface markers (P<0.01). There were more cells that expressed SSEA1 and CD117 in DMEM-KO with 20% FBS compared toRPMI-1640 (Fig .3,P<0.01). The population of mESCs that expressed SSEA1 and CD117 that were cultured on PCL in DMEM-KO with 20% FBS were higher than the other conditions. 

**Fig.4:**
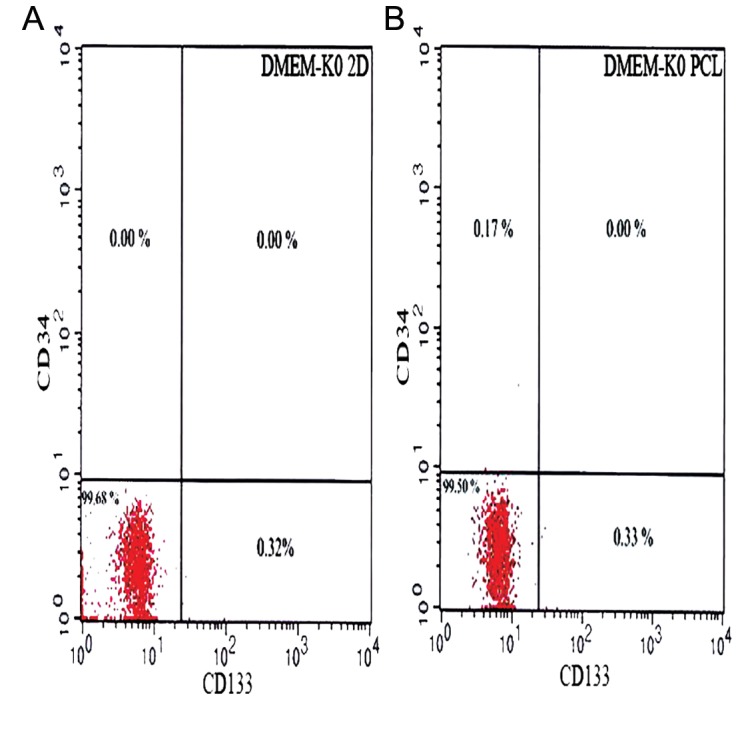
Flowcytometric analysis of mouse embryonic stem cells (mESCs) in different culture conditions prior to differentiation. Flowcytometric analysis was performed for the specific markers of hematopoietic stem cells (HSCs) using CD34-PE and CD133- FITC antibodies. A. Cell population that expressed CD34 and CD133 in knockout Dulbecco’s modified eagle medium (DMEMKO) tissue culture plate (TCP) and B. Cell population that expressed CD34 and CD133 in DMEM-KO polycaprolactone (PCL).

### Comparison of mouse hematopoietic stem cell population that expressed surface markers including CD34 and CD133 in polycaprolactone and tissue culture plate conditions after 7 days of differentiation along cytokines treatment 

**Fig.5:**
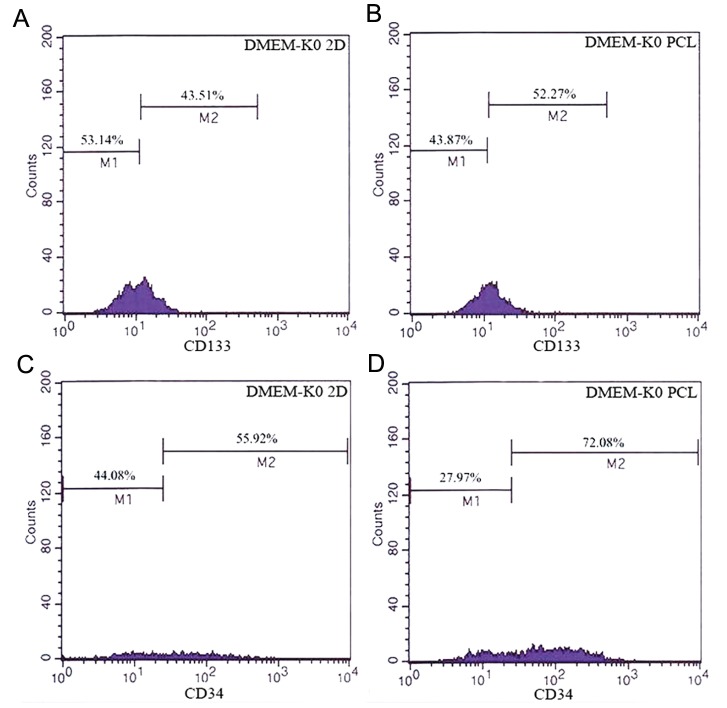
Flowcytometric analysis of mouse hematopoietic stem cells (mHSCs) in different culture conditions after differentiation. Flowcytometric analysis was performed for the specific markers of mHSCs using CD133-FITC and CD34-PE antibodies. A. CD133 expression histogram in tissue culture plate (TCP)+hematopoietic induction medium, B. CD133 expression histogram in polycaprolactone (PCL)+hematopoietic induction medium, C. CD34 expression histogram in TCP+hematopoietic induction medium and D. CD34 expression histogram in PCL+hematopoietic induction medium.

We transferred 7-day old EBs to gelatin-coated 24-well plates. Transferred EBs were maintained in IMDM with 30% FBS for 2 days. During the differentiation stage, EBs were reseeded onto PCL and TCP with differentiation media that contained IMDM-30% FBS supplemented with SCF (20 ng/ ml), IL-3 (20 ng/ml), IL-6 (2 ng/ml) and FL (20 ng/ ml) cytokines for 7 days. Cell surface marker expression analysis indicated that cells which expressed CD34 and CD133 under IMDM PCL conditions was significantly higher than IMDM TCP (Fig.5, P<0.01). We found that the cell population which expressed CD133 marker in hematopoietic induction medium on PCL was 52.27% compared to 43.51% in TCP. In parallel, 72.08% of cells expressed CD34 in IMDM PCL compared to 55.92% for IMDM TCP. 

## Discussion

Biodegradable and biocompatible scaffolds should mimic the biological, chemical and physical function of the ECM as much as possible. The ECM provides a substrate with specific ligands for cell adhesion and migration, in addition to regulation of cell proliferation, survival and differentiation by providing various growth factors. The nanofibrous structure of the ECM provides a 3D space and appropriate volume to cells for attachment and expansion (38). The ECM is believed to provide an ES 'niche' and plays a major role in ES renewal and pluripotency (39). The colonization and maintenance of ESCs in an undifferentiated state using various biomaterials have been described in several reports (40,42). 

In order to support cell growth, a scaffold must closely mimic the ECM in structure and function. The nanofiber is considered a most useful structure among scaffolds. Nanofibers are expected to overcome the limitations of TCP and feeder layers. Numerous advantages of nanofibers include a high surface area per unit volume, numerous fibers in the unit area, high porosity, a micro space created between fibers, flexibility, and biodegradable nature. Nanofibers can be produced through phase separation, self-assembly and electrospinning methods, among others. The nanofiber, as a scaffold, should provide an appropriate environment as collagen of the ECM for tissue engineering (38). 

Recently, the 3D culturing method has been developed for culturing various cell types, including mESCs. In general, 3D scaffolds can support high cell densities and are advantageous for use as a tissue supporting environment (43). 

Among various nanofiber polymers, PCL is an aliphatic polyester generally used in pharmaceutical products and is considered to be a non-toxic, biocompatible material. Brodbeck et al. (44) have recently demonstrated that the hydrophilicity of the substrate surface could have an impact on apoptosis as the hydrophilic surface produced a decreased expression of pro-inflammatory cytokines. Yoshimoto et al. (45) demonstrated that electrospun PCL was a promising candidate scaffold for bone tissue engineering. Studies showed that PCL scaffolds could support a wide variety of cell types such as muscle cells, mesenchymal stem cells (MSCs), glia cells, and chondrocytes (45,46). 

Recently, it has been demonstrated that the addition of glutaraldehyde into PCL not only reduced the potential cytotoxicity that this chemical crosslinking reagent could cause, but it also produced a new composite with improved mechanical and biological properties (47,50). Electrospinning is the most widely used technique to create fibrous structures that have favorable mechanical and biological properties (51). Electrospun nanofibers have been incorporated in stem cell cultures to provide a desired microenvironment for their growth and differentiation, and to ultimately mimic the SC niche (52). 

In the present study, we analyzed the maintenance of stemness and pluripotency of mESCs cultured on PCL and TCP using DMEM-KO and RPMI-1640 supplemented with 20% FBS. The first aim of the study was to establish optimum culture conditions for mESCs maintenance and expansion in order to maintain these cells for numerous passages without compromising quality, loss of characteristics and functionality of cell surface markers such as SSEA1 and CD117. The results showed significantly more mESCs cultured on PCL in DMEM-KO compared to mESCs cultured on RPMI-1640 under the same culture conditions after 48 and 96 hours. Our results showed that mESCs cultured on TCP supplemented with DMEM-KO were significantly higher than mESCs cultured on TCP supplemented with RPMI-1640 after 48 and 96 hours, but they were not higher than mESCs cultured on PCL. 

Based on our results, DMEM-KO had better performance to improve the morphology and proliferation of ESCs compared to RPMI-1640. DMEMKO has reduced osmolarity to mimic the natural environment of embryonic tissue and higher glucose levels compared to other DMEM media and RPMI-1640, which result in improved cell morphology and reduced cell differentiation (53). 

The proliferation rate of mESCs on PCL electrospun nanofibrous scaffolds was significantly higher than TCP after 48 and 96 hours. mESC culture efficiency has not been previously tested on PCL, however, Hashemia et al. (54) showed that the proliferation rate of mESCs on polyethersulfone (PES) nanofiber treated with collagen was significantly higher than PES nanofiber after 96 hours. Research has demonstrated that mechanical signals are transduced to the cell cytoskeleton through the activation of Rho, a small GTPase, and Rho kinase (55). 

Other studies reported that enhanced proliferation and self-renewal of mESCs on synthetic polyamide matrix (Ultra-Web) correlated with both the activation of the small GTPaseRac and phosphoinositide3-kinase (PI3K) pathways. The pathways have been recently reported to promote self-renewal in mESCs (56,58). However the signaling pathways involved in supporting mESCs growth on PCL are unknown and should be clarified. 

Various studies have shown that the nanofibrous scaffolds can significantly influence the proliferation rate of various cell types (59,62). Liu et al. (63) showed that hESCs proliferation was higher in both fibrin and PEGylated fibrin gels versus TCP and methylcellulose controls (63). 

In addition, our results revealed that the mESCs cultured for 48 and 96 hours on PCL and TCP had typical undifferentiated morphology and enhanced proliferation. They also showed similar continued expression of stemness and pluripotency associated marker expression which included SSEA-1 and CD117. Our results showed some improvement in these characteristics in mESCs cultured for 48 and 96 hours on PCL compared with TCP. These results could be partially compared to the findings of Hashemia et al. in which the percentages of Oct-4, SSEA-1, and ALP-positive colonies on PES nanofiber treated with collagen have significantly increased in comparison with PES nanofibers (PES) and the gelatin coated plate although the molecular composition of PES and PCL totally differ (54). These *in vitro* findings suggested that PCL maintained the pluripotency and other specific characteristics of mESCs in comparison with other conditions. 

The specific mechanism by which nanofibrous scaffolds support self-renewal of mESCs is not entirely clear. It has been shown that electrospun polyamide nanofibers (Ultra-Web) can promote proliferation and self-renewal of mESCs through mechanisms that involve Rac, PI3K/AKT signaling pathways, and up-regulation of Nanog and c-Fos (58). 

In this study, we evaluated CD34 and CD133 cell surface markers in SSEA1+/CD117+ cell populations to establish that both PCL and TCP conditions did not impact differentiation of mESCs. Our results have shown that when mESC were cultured under PCL and TCP conditions, there was no significant differentiation into mHSCs. Differentiation of ESCs does not only depend on the presence of the proper molecular stimuli provided by the feeder layers and cytokine cocktails, but also on the specific physical conditions under which the ESCs are cultured. 3D EB differentiation cultures are based on the ability of differentiating ESCs to spontaneously generate various cell types including those that support hematopoietic development. 

The second purpose of study was to evaluate the differentiation quality of mESCs into mHSCs under PCL and TCP conditions. To our knowledge, this was the first study that used PCL to maintain pluripotency of ESCs *in vitro* and their differentiation into mHSCs. We found that after induction of hematopoietic differentiation, the cell population that expressed CD34 and CD133 markers on PCL was significantly higher than TCP. ES cell differentiation has been demonstrated to be strongly affected by interactions with external physical and chemical stimuli, including the topography and composition of the ECM (64,65). However this study has shown the supporting effect of PCL on hematopoietic differentiation of mESCs. The 3D method for hematopoietic differentiation of ESCs was advantageous over the adherent ESC differentiation cultures on a gelatinized surface that had significantly reduced hematopoietic development (66). Interestingly, the frequency of generation of hematopoietic progenitors in different 3D methods form EB cultures (liquid suspension, methylcellulose and hanging drop) was similar (35). In particular, physical and mechanical properties of the 3D microenvironment, such as smaller scaffold pore size and higher polymer concentration, resulted in significantly enhanced hematopoiesis (67). 

## Conclusion

We analyzed the effect of nanofiber scaffolds on survival and proliferation of mESCs as well as differentiation into mHSCs compared with gelatin coated TCP. The results showed that the nanofiber scaffold was an effective surface for improved survival and differentiation of mESCs into mHSCs compared with gelatin coated TCP. Thus the viability and proliferation of mESCs as well as differentiation into mHSCs have been influenced by nanofibrous scaffolds. More in depth studies are necessary to understand how the topographical features of electrospun fibers affect cell growth and behavior. This will be achieved by designing biomimetic scaffolds for tissue engineering. 
